# Editorial: Role of estrogens as key regulators of energy homeostasis

**DOI:** 10.3389/fendo.2023.1279619

**Published:** 2023-09-06

**Authors:** Marta G. Novelle, Alberto Camacho-Morales, Pablo B. Martínez De Morentin

**Affiliations:** ^1^ Department of Genetics, Physiology, and Microbiology (Unit of Animal Physiology), Faculty of Biology, Complutense University, Madrid, Spain; ^2^ College of Medicine, Biochemistry and Molecular Medicine Department, Universidad Autónoma de Nuevo León, Monterrey, Mexico; ^3^ School of Biomedical Sciences, Faculty of Biological Sciences, University of Leeds, Leeds, United Kingdom; ^4^ The Rowett Institute, University of Aberdeen, Aberdeen, United Kingdom

**Keywords:** energy homeostasis, estrogens, neuroregulation, sex hormones, obesity

Over the last few decades, extensive research has revealed the metabolic importance of sex hormones and sex-specific molecular mechanisms beyond their role in reproduction. Strong evidence supports sexual dimorphism in homeostatic responses and in the incidence and evolution of metabolic diseases. Indeed, some health conditions have been shown to have a worse prognosis in women. Sex hormones play a critical role in shaping the molecular and cellular fate, by modulating their gene expression, cell-cycle, and even the pharmacokinetics of specific drugs. Understanding these mechanisms is crucial to design new therapies and sex-specific interventions to improve the effectiveness of healthcare while more efforts need to be directed towards drawing a more comprehensive description of the metabolic differences in both sexes.

In this context, estrogens, the primary sex hormones in females, and their interactions with estrogen receptors, are crucial for the onset, prevalence, and severity of metabolic diseases. Similarly, estrogens are associated with specific clinical implications and treatment outcomes. Despite the ongoing efforts to fill knowledge gaps, gaining a deeper understanding of estrogen’s influence on energy balance remains imperative.

Here, we present a Research Topic containing comprehensive reviews on how estrogens regulate energy homeostasis, acting at both central and peripheral levels. As a result, estrogen signaling pathways emerge as promising therapeutic targets to mitigate metabolic disturbances, such as obesity and its associated comorbidities. In the context of this Research Topic, Vigil et al. draw attention to the escalating problem of obesity in women worldwide, significantly impacting their health and lifespan. The research has demonstrated that while premenopausal women exhibit lower incidences of metabolic disruptions due to estrogen’s protective effects, these benefits diminish following menopause. Comparable results have been observed during other stages of a woman’s life when estradiol levels are low. Conversely, conditions such as pregnancy, characterized by high estradiol levels, pose a high-risk period where susceptible women are more prone to developing metabolic comorbidities like obesity and gestational diabetes. In their review, the authors explore the mechanisms governing the actions of estradiol during the menstrual cycle on food intake and energy expenditure. Besides, they describe how estradiol interacts with other peripheral feedback signals that control appetite and energy expenditure, particularly focusing on peripheral mediators like glucagon-like peptide-1 (GLP-1). They highlight how a decline or imbalance in estradiol levels can affect insulin sensitivity in the brain and potentially contribute to the development of conditions like Parkinson’s or Alzheimer’s disease, considering future applications of estradiol and GLP-1 conjugates in protecting against cerebral insulin resistance and the resulting neurodegenerative disorders. In addition to the metabolic effects, authors emphasize the association between steroid hormones and women’s mental health as a defining factor when considering therapeutic approaches.

Estrogens are potent regulators of body temperature and Fernández-Peña et al. provide a thorough review on the differences behind the mechanisms regulating temperature in male and female, and their influence on food intake, body weight, energy expenditure and ultimately, the impact in body composition. The authors delve into a detailed exploration of the pathways involved in sex differences in thermosensation and thermoregulation, shedding light on the variations in the main thermoregulatory mechanisms. They cover a broad range of aspects, from heat-conserving and heat-dissipating mechanisms to the thermogenic mechanism in brown adipose tissue (BAT). They emphasize the close link between regulation of temperature, and energy and how the molecular mechanisms in charge of sensing environmental temperature are intrinsic part of the circuits regulating energy homeostasis and therefore, metabolism. In addition, the authors provide a critical view on the flaws of experimental designs and their translational significance, reiterating the need of factoring sex as a variable when studying molecular pathways and metabolism, in both laboratory animals and human trials. This more inclusive approach will allow for a more nuanced interpretation of the results and ensure that therapeutic interventions and treatments can be tailored to address the unique needs and responses of each sex.

Abundant clinical and epidemiological studies have consistently revealed significant differences between men and women in various aspects of lipid metabolism. These differences encompass body weight, body composition, white adipose tissue (WAT) accumulation and distribution, and metabolic activity in brown adipose tissue. Evidence strongly suggests that estrogens play a crucial role in shaping lipid metabolism through their influence on specific brain regions. Understanding the interplay between sex hormones, brain regions, and adipose tissue metabolism is essential for comprehending the underlying mechanisms responsible for the disparities observed. Irizarry et al. review the role of the hypothalamus, specifically the ventromedial hypothalamus (VMH), as a key brain region mediating the metabolic effects of estrogens. They walk through the mechanism of action of the two major estrogen receptors (ERα and ERβ), as well as other novel receptors like the G protein-coupled estrogen receptor 1 (GPER1) and Gq protein-coupled receptor (Gq-mER), both of which represent promising therapeutic targets for metabolic syndrome. Furthermore, the researchers explore the interaction of the estrogen pathway with peripheral hormones, adipokines, and key regulators of cell metabolism, namely mTOR (Mammalian Target of Rapamycin) and AMPK (AMP-activated protein kinase). This critical analysis of the current data helps unravel the intricate connections between hormonal pathways and metabolic regulation, contributing to a better understanding of the mechanisms driving sex-based disparities in adipose physiology.

While there is a vast amount of information highlighting the positive impact of estrogens on various metabolic areas, Lizcano reminds us of that hormone replacement therapy (HRT) containing estrogens, initially designed to relieve the symptoms of menopause, remains not risk-free, requiring careful consideration of the associated risks and benefits. Although HRT can effectively improve metabolic disorders linked to an estrogen deficiency, such as reducing the risk of coronary disease, cerebrovascular accidents, obesity, and type 2 diabetes mellitus; the patient individual metabolic profile, including existing health conditions and risk factors, is decisive for the prescription of HRT. Accordingly, it is also essential to decipher the role of new synthetic compounds with estrogenic effects in improving metabolic disturbances associated with estrogen deficiency, and to consider the impact of environmental pollutants that can mimic hormonal activity, thereby interfering with many physiological systems by disrupting the hormonal regulation.

Endocrine-disrupting chemicals (EDCs) can interfere with estrogen receptors in the body, as their chemical structure closely resembles that of estradiol. Among these, bisphenol A (BPA) is one of the most extensively studied chemicals, commonly used in the manufacturing of plastics for various everyday items. Due to its estrogen-like activity, BPA has been banned or voluntarily removed from the food supply chain in several countries. As a result, alternative bisphenols like bisphenol S (BPS) have been introduced. However, there is an ongoing debate surrounding their safety as well. Considering this concern, Téteau et al. have taken the initiative to investigate the potential impact of BPS as a possible endocrine disruptor of secreted steroid hormones, their precursors, and metabolites in mammals. The study utilized ewes as an animal model, which serves as a relevant model for investigating female reproduction and toxicology related to bisphenols. Their research has proven to be of significance as it sheds light on the consequences of exposure to BPS at doses previously established as guidelines for BPA in Europe. Remarkably, they observed that this exposure influenced the concentration of steroid content in the preovulatory follicle, oviduct fluid and plasma. Interestingly, the effects of BPS were not uniform across the fluids, suggesting that BPS may have specific effects depending on the compartment being analyzed. Notably, estradiol appeared to be one of the most affected steroids, highlighting the estrogenic effects of BPS. Additionally, the researchers found that the metabolic status of the ewes played a significant role in modulating the effects of BPS. Ewes with higher fat content were more sensitive to the effects of this endocrine disruptor, indicating that physiological factors can influence the response to BPS exposure. This information can contribute to better risk assessments and informed decision-making regarding the use and regulation of bisphenols in everyday products, ultimately safeguarding human and environmental health.

Overall, this Research Topic provides an updated view of the knowledge of the role of estrogens as key elements in the regulation of energy homeostasis. The comprehensive studies presented here provide valuable insights into the intricate mechanisms through which estrogens influence metabolic processes. The information compiled in this Research Topic will undoubtedly pave the way for innovative strategies to improve human health and well-being.

## Author contributions

MN: Writing – original draft, Writing – review & editing. AC-M: Writing – original draft, Writing – review & editing. PM: Writing – original draft, Writing – review & editing.

